# CD38 Exacerbates Focal Cytokine Production, Postischemic Inflammation and Brain Injury after Focal Cerebral Ischemia

**DOI:** 10.1371/journal.pone.0019046

**Published:** 2011-05-13

**Authors:** Chi-un Choe, Kerstin Lardong, Mathias Gelderblom, Peter Ludewig, Frank Leypoldt, Friedrich Koch-Nolte, Christian Gerloff, Tim Magnus

**Affiliations:** 1 Department of Neurology, University Hospital Hamburg-Eppendorf, Hamburg, Germany; 2 Department of Immunology, University Hospital Hamburg-Eppendorf, Hamburg, Germany; 3 Department of Clinical Chemistry, University Hospital Hamburg-Eppendorf, Hamburg, Germany; Julius-Maximilians-Universität Würzburg, Germany

## Abstract

**Background:**

Converging evidence suggests that inflammatory processes significantly influence brain injury and clinical impairment in ischemic stroke. Although early studies suggested a key role of lymphocytes, recent data has emphasized the orchestrating function of innate immunity, i.e., macrophages and microglia. The bifunctional receptor and ectoenzyme CD38 synthesizes calcium-mobilizing second messengers (e.g., cyclic ADP-ribose), which have been shown to be necessary for activation and migration of myeloid immune cells. Therefore, we investigated the dynamics of CD38 in stroke and the impact of CD38-deficiency on cytokine production, inflammation and cerebral damage in a mouse model of cerebral ischemia-reperfusion.

**Methodology/Principal Findings:**

We show that the local expression of the chemokine MCP-1 was attenuated in CD38-deficient mice compared with wildtype mice after focal cerebral ischemia and reperfusion. In contrast, no significant induction of MCP-1 expression was observed in peripheral blood after 6 hours. Flow cytometry analysis revealed less infiltrating macrophages and lymphocytes in the ischemic hemisphere of CD38-deficient mice, whereas the amount of resident microglia was unaltered. An up-regulation of CD38 expression was observed in macrophages and CD8^+^ cells after focal cerebral ischemia in wildtype mice, whereas CD38 expression was unchanged in microglia. Finally, we demonstrate that CD38-deficiency decreases the cerebral ischemic injury and the persistent neurological deficit after three days of reperfusion in this murine temporary middle cerebral artery occlusion (tMCAO) model.

**Conclusion/Significance:**

CD38 is differentially regulated following stroke and its deficiency attenuates the postischemic chemokine production, the immune cell infiltration and the cerebral injury after temporary ischemia and reperfusion. Therefore CD38 might prove a therapeutic target in ischemic stroke.

## Introduction

Inflammation plays an important role in infections to fight pathogens, but can be detrimental in autoimmune diseases and ischemia-reperfusion injury. In stroke, inflammatory cascades are triggered by cerebral ischemia-reperfusion and considerably influence secondary brain injury due to cytotoxic neuronal cell death [1]. The activation, migration and accumulation of immune cells characterize crucial steps of postischemic inflammation and are mediated by extracellular cytokines (e.g. chemokines) as well as intracellular molecular signals (e.g. second messengers) [2]. Although different chemokines and receptors, like monocyte chemotactic protein-1 (MCP-1 or CCL2) and CCR2, are involved in immune cell activation and chemotaxis after stroke, an approach aiming at multiple targets remains to be proven [3], [4].

The bifunctional ectoenzyme and receptor CD38 represents such a versatile modulator regulating the migration of neutrophils and dendritic cells in response to different chemokines, like CCL2, CCL19, CCL21, CXCL12, as well as to bacterial-derived chemoattractants, like N-formyl-methione-leucine-phenylalanine (fMLF) [5], [6], [7], [8]. CD38, which is widely expressed on hematopoetic as well as non-hematopoetic cells, catalyzes the production of second messengers (i.e cyclic adenosine dinucleotide phosphoribose (cADPR), adenosine dinucleotide phosphoribose (ADPR), nicotinic acid adenine dinucleotide phosphate (NAADP)), which act as potent intracellular calcium mobilizing agents [5], [6], [7], [8]. In addition to its enzymatic function, CD38 has been suggested to act as a receptor interacting with CD31 on endothelial cells to sustain adhesion and rolling of lymphocytes. These functions of CD38 as ectoenzyme and receptor have been implicated to mediate the humoral and innate immune response of lymphoid and myeloid lineage cells [5], [9]. Especially, the priming of T-cells as well as the directional migration of myeloid lineage cells seem to depend on CD38 and its products ADPR and cADPR [5], [10].

Just recently modulation of mainly detrimental inflammatory processes have been attributed to hematopoetic cells like lymphocytes and macrophages, which are the primary source for pro-inflammatory cytokines like IL-17 and IL-23, respectively [11], [12]. Therefore, the infiltration of the ischemic brain by hematopoetic immune cells marks an essential step of postischemic inflammation. CD38-deficiency has been demonstrated to impair migration of hematopoetic myeloid cells and to attenuate lymphocyte activation. To determine the functional significance of CD38 in ischemic stroke, we compared local and systemic cytokine and chemokine levels, the amount of infiltrating inflammatory cells, the CD38 expression pattern of leukocyte subpopulations, and infarct sizes in wildtype and CD38-deficient mice.

## Materials and Methods

### Mice

C57BL/6 mice and GFP transgenic mice (TgN(TIE2GFP)287Sato/J in FVB/N/J background) were obtained from the Jackson Laboratory (Bar Harbor, ME). CD38^−/−^ mice were backcrossed onto the C57BL/6 background for 12 generations as described previously [5]. All animal experiments have been conducted according to relevant national and international guidelines (German Animal Welfare Act) and have been approved by the local Animal Care and Use Committee (Behoerde fuer Soziales, Familie, Gesundheit und Verbraucherschutz - Lebensmittelsicherheit und Veterinaerwesen – animal permit number G08/014_CD38).

### Temporary Middle Cerebral Artery Occlusion (tMCAO)

C57BL/6 male mice (12–15 weeks) were used in accordance with the Guide for the Care and Use of Laboratory Animals and approved by the Institutional Animal Care and Use. Briefly, mice were anesthetized (isoflurane 1–2% v/v oxygen) and analgesized (buprenorphine 0.03 mg/kg b.w. i.p. every 12 h for 24 h). As previously described, tMCAO was achieved by using the intraluminal filament method (6–0 nylon) for one hour [13], [14]. Exemplary mice were monitored using transcranial temporal laser Doppler and every mouse was scored on a scale from 0–5 (0 no deficit, 1 preferential turning, 2 circling, 3 longitudinal rolling, 4 no movement, 5 death) after reawakening and daily until sacrifice. Mice were sacrificed at indicated time points after reperfusion using isoflurane and decapitation. To measure the infarct volume, brains were removed after MCAO and evaluated using 2,3,5-triphenyltetrazolium chloride staining of 1 mm thick brain slices. All analyzed infarcts involved cortical tissue. The surgical procedure and the analysis were conducted in a blinded fashion. The stained sections were scanned (2.400 dpi), the digitized images of each brain section and the infarct area were quantified using a computerized image analysis program (Image J, USA).

### Bone Marrow Transplantation

Bone marrow chimeric mice were created as described previously (Cassiani-Ingoni et al., 2007). In brief, 6 weeks old male congenic C57BL/6J-recipients (20–30 g) were sublethally irradiated with 6.5 Gy. Bone marrow was obtained by flushing the femur bones from male donor GFP-transgenic animals (C57BL/6J) with sterile phosphate buffered saline. Bone marrow cells were suspended in the same buffer, washed several times, counted and injected into the tail vein at 1×10^7^ cells/recipient. Animals with more than 90% CD45^+^ GFP^+^ leukocytes verified by peripheral blood analysis were used for further experiments 6 weeks after transplantation.

### Mouse Brain Extracts

Animals were euthanized 6 or 24 hours postreperfusion and brains were directly prepared in cold PBS. Hemispheres were separately homogenised mechanically in homogenisation buffer (140 mM NaCl; 20 mM Tris-HCl pH 7,6; 5 mM EDTA; 1× complete protease inhibitors) and extracts additionally sonicated for 30 s. Resulting extracts were centrifuged (6000 rpm) for 10 min at 4°C. Supernatants were further centrifuged (14000 rpm) for 10 min at 4°C and final supernatants used for ELISA analysis.

### Cytokine Production (ELISA)

For quantitative determination of MCP-1, TNF-α and IFN-γ protein level in serum or brain extracts, standard ELISA was performed according to the manufacturer's instructions (ELISA Set, OptEIA™, BD Biosciences, Heidelberg, Germany). Optical density at 450 nm was obtained using the Tecan Sunrise™ and Magellan™ system (Maennedorf, Austria) and protein concentrations were determined using a provided standard curve.

### Fluorescence-Activated Cell Sorter (FACS) Analysis

As previously described, animals were euthanized and perfused with PBS [14]. Brains were dissected, cerebella removed, and hemispheres divided into left ischemic (ipsilesional) or right nonischemic (contralesional). Three to 4 hemispheres were pooled, incubated for 30 minutes at 37°C (1 mg/ml collagenase, 0.1 mg/ml DNAse I in DMEM), and pressed through a cell strainer (40 µm; BD Biosciences, Heidelberg, Germany). Next, cells were incubated with standard erythrocyte lysis buffer on ice, separated from myelin and debris by Percoll gradient (GE Healthcare; 1095 g/ml and 1030 g/ml) centrifugation, and incubated with appropriate antibody cocktails as previously described [14] (30 minutes, room temperature) in fluorescence-activated cell sorter buffer (0.5% bovine serum albumin, 0.02% sodium azide in phosphatebuffered saline). For TrueCount (Becton Dickinson) fluorescence bead absolute quantification, 10% of cells were quantified according to the protocol on the basis of CD45-positive events. For CD38 expression cells were labelled with anti-mouse fluorochrome-conjugated antibodies (BD Biosciences): CD38-PE (1∶300), CD11b-FITC (1∶300), CD11c-APC (1∶300), CD45-PE-Cy7 (1∶150), CD8-Pacific Blue (1∶300) and CD4-APC-Cy7 (1∶150) and were distinguished as previously described (figure S1) [14] Cells were analyzed by blinded evaluators using a LSR II (BD Biosciences) and FACS Diva software (BD Biosciences). Up to 2 000 000 forward light scatter events per tube were acquired.

### Immunohistochemistry

Mice were anesthetized with isoflurane inhalant and perfused through the left ventricle using 10ml PBS followed by 50ml of ice-cold 4% PFA. The brains were post-fixed in 4% PFA overnight at 4°C followed by cryoprotection in 30% sucrose (w/v) in PBS until the brains sank to the bottom of the solution. After snap freezing of the embedded brains in isopentane pre-cooled with liquid nitrogen, brains were cut into 6 µm frontal sections using a Leica CM3050. Sections were then exposed to PBS containing 0.3% Triton X-100 and 10% normal goat serum to block nonspecific antibody binding for a minimum of 60 min, followed by incubation with the primary antibodies and lectins at 4°C overnight (CD11b, 1∶100, BD Pharmingen; GFAP, 1∶1000, DAKO; isolectin GS IB4 Alexa Fluor 488, 1∶500, Invitrogen). Sections were then incubated with the appropriate secondary antibodies (Alexa Fluor 546 goat anti-rat IgG, Alexa Fluor 647 Goat Anti-Rabbit, Invitrogen) at room temperature for 1h, and counterstained with DAPI (1∶1000, Invitrogen). Negative control sections from each animal were obtained by omitting primary antibodies. Stained sections were examined with a Leica DM5000B microscope.

### Statistical Analyses

Data in text and figures are mean ± SEM for the indicated number (n) of animals or cultures. Statistical comparisons were made by student's t-test for infarct size, neurological score and FACS-analysis. Significance was set at p<0.05. All statistical analyses were performed using GraphPad Prism software (GraphPad Software, Inc.).

## Results

### Cytokine and Chemokine Levels After tMCAO

**Figure 1:**
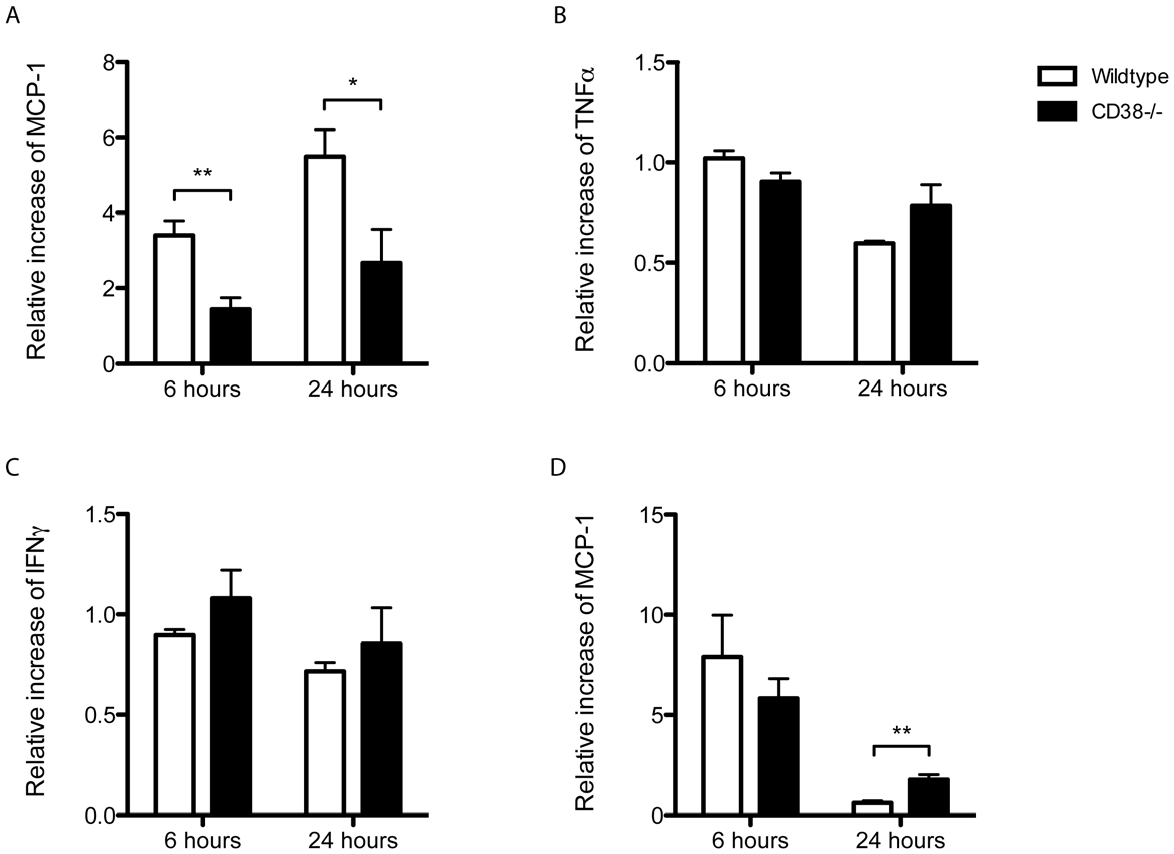
CD38^−/−^ mice produce less chemokine MCP-1 after tMCAO than wildytpe mice. Ipsilateral hemispheres were lysed 6 or 24 hours after tMCAO and concentrations of MCP-1, TNF-α and IFN-γ were determined by ELISA. Results are represented as increase in cytokine levels relative to sham-operated control animals. Control values of wildtype and CD38^−/−^ mice did not differ significantly from each other (A). In CD38^−/−^ mice levels of MCP-1 increased to a lesser extend than in wildtype mice after 6 and 24 hours reperfusion, whereas cytokine levels of TNF-α and IFN-γ stayed unaltered (B, C). MCP-1 concentrations in peripheral blood (D) did not differ after 6 hours (n = 5–8; *p<0.05; **p<0.01; ***p<0.001 versus control assessed by one-sample t-test).

We used the tMCAO model to explore the role of CD38 in postischemic chemoattraction. Focal cerebral ischemia was induced for one hour with the intraluminal filament technique in wildtype and CD38^−/−^ animals. The initial neurological impairment did not differ between genotypes indicating a similar brain lesion. Six and 24 hours after tMCAO, the ipsilateral hemispheres were prepared and protein lysates were analysed for cytokines with ELISA. Both, wildtype and CD38^−/−^ displayed increased levels of MCP-1, but the increase was significantly lower in CD38^−/−^ mice at 6 and 24 hours after tMCAO compared with wildtype mice (wildtype vs CD38^−/−^ 3,4±0,4 vs 1,5±0,3 at 6h and 5,5±0,7 vs 2,7±0,9 at 24h, n = 5–7, p<0.05, figure 1A), while TNF-α and IFN-γ concentrations in the brain were unaltered throughout this time period (wildtype vs CD38^−/−^ for TNF-α 1,0±0,04 vs 0,9±0,04 at 6h and 0,6±0,01 vs 0,8±0,1 at 24h, n = 5–8; for INF-γ 0,9±0,03 vs 1,1±0,1 at 6h and 0,7±0,04 vs 0,9±0,2 at 24h, n = 5–8, p>0.05, figure 1B, C). MCP-1 levels were strongly increased also in peripheral blood of wildtype and CD38^−/−^ mice, albeit to similar extents at six hours after tMCAO (wildtype vs CD38^−/−^ 7,9±2,1 vs 5,8±1,0 at 6h and 0,6±0,1 vs 1,8±0,2 at 24h, n = 5–8, p>0.05, figure 1D). Twenty-four hours after tMCAO, MCP-1 levels in peripheral blood were even higher in CD38^−/−^ mice compared with wildtype animals (p<0.01, figure 1D).

### Postischemic Inflammatory Response

**Figure 2:**
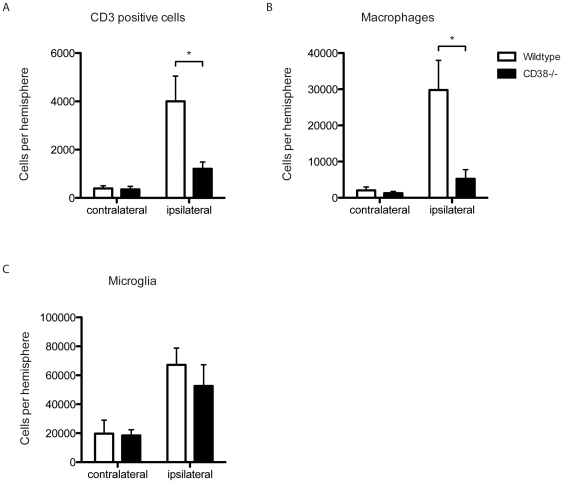
CD38 modulates cerebral postischemic immune cell infiltration. Infiltrating immune cells were isolated after tMCAO from wildtype and CD38^−/−^ brains and subdifferentiated by multi-color flow cytometry. Significantly less CD3^+^ cells and macrophages were found in the ischemic hemisphere of CD38^−/−^ mice (A, B), whereas the number of microglia was unaltered (C) (n = 5–6, *p<0.05). The amount of immune cells, i.e. CD3^+^ cells, macrophages and microglia, did not change in the contralateral control hemisphere (n = 5–6; p>0.05).

Focal chemokine production differentially influences directional migration of hematopoetic inflammatory cells. MCP-1 represents a well-characterized pro-inflammatory chemokine, which attracts immune cells of myeloid origin, especially monocytes to the tissue with MCP-1 accumulation. Therefore, immune cell subtypes invading the ischemic hemisphere were characterized by FACS-analysis after one hour of middle cerebral artery occlusion and three days of reperfusion (figure S1). Whilst the accumulation of CD3^+^ cells and macrophages in the ischemic hemisphere was significantly decreased in CD38^−/−^ compared with wildtype mice (wildtype vs CD38^−/−^: CD3^+^ cells 1275±215 vs 638±127 and macrophages 29782±8196 vs 5200±2541, n = 3 experiments with 3–4 animals each, p<0.05, figure 2A, B), the amount of microglia did not differ significantly (wildtype vs CD38^−/−^: 67189±1160 vs 52648±1462, n = 3 experiments with 3–4 animals each, p>0.05, figure 2C). Results from FACS-experiments corresponded well to immunohistochemical studies, which revealed smaller infarct sizes after staining for GFAP and reduced amounts of macrophages, which were distinguished by positive staining for CD11b and isolectin as well as cell morphology (figure S2).

In order to distinguish between hematopoetic and local immune cells, we used GFP bone marrow chimera. Irradiated wildtype mice reconstituted with GFP positive bone marrow cells were subjected 6 weeks after transplantation to one hour of tMCAO and three days of reperfusion. Immune cell subtypes localized in the ischemic hemisphere were discriminated by FACS-analysis (figure S3). Macrophages (dark grey) and myeloid dendritic cells (light grey) highly expressed GFP and therefore originated from the bone marrow. In contrast, resident microglia were GFP negative (black).

### CD38 Expression of Immune Cells

**Figure 3:**
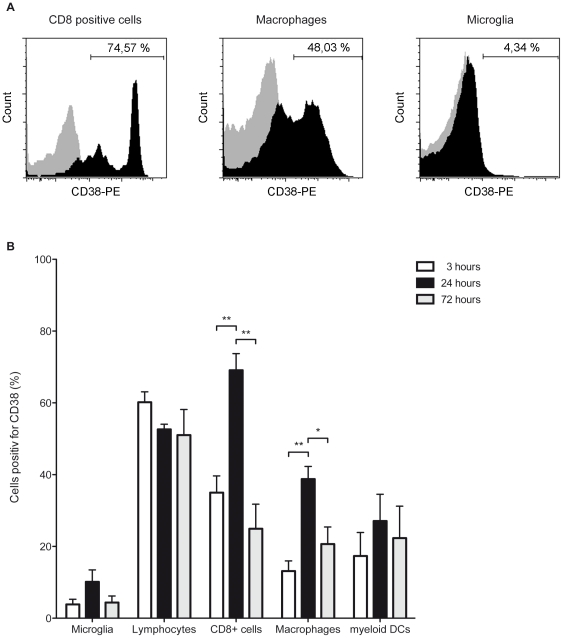
Infiltrating immune cells express CD38. Immune cells infiltrating the ischemic brain of wildtype mice were isolated after tMCAO and subpopulations were analysed by multi-color flow cytometry. The amount of CD38^+^ expressing CD8^+^ cells (left) and macrophages (middle) significantly increases 24 hours after tMCAO, whereas the subpopulation of CD38^+^ microglia (right) stays unaltered (A). After 72 hours, the number of CD38^+^ CD8^+^ cells and CD38^+^ macrophages decreased to 3 hour values (n = 3–4 in each group; *p<0.05 and **p<0.01).

Upon activation, mature lymphocytes and microglia have been demonstrated to up-regulate CD38 expression [15], [16]. Furthermore, CD38 is known to orchestrate the migration of myeloid immune cells and modulate the cytokine production of lymphocytes [16], [17]). Therefore, we analyzed CD38 expression on immune cells accumulating in the ischemic brain 3, 24 and 72 hours after focal cerebral ischemia induction in CD38^−/−^ and wildtype mice. The amount of CD38^high^ microglia, CD38^high^ CD3^+^ cells and CD38^high^ myeloid dendritic cells did not change significantly during the observed reperfusion period (p>0.05, figure 3). In contrast, the subpopulation of CD38^high^ macrophages increased from 13±2.8% three hours after tMCAO to 38±3.5% twenty-four hours after tMCAO, but normalized to 21±4.8% already 72 hours after tMCAO (3 hours vs 24 hours, p<0.01; 24 hours vs 72 hours, p<0.05; 3 hours vs 72 hours p>0.05; figure 3A middle and B). Similarly, the fraction of CD38^high^ CD8^+^ cells increased from 35±4.6% three hours after tMCAO to 69±4.6% twenty-four hours after tMCAO and normalized to 24.9±6.8% three days after focal cerebral ischemia (3 hours vs 24 hours, p<0.01; 24 hours vs 72 hours, p<0.01; 3 hours vs 72 hours p>0.05; figure 3B).

### CD38 Increases Focal Ischemic Injury After Transient Middle Cerebral Artery Occlusion

**Figure 4:**
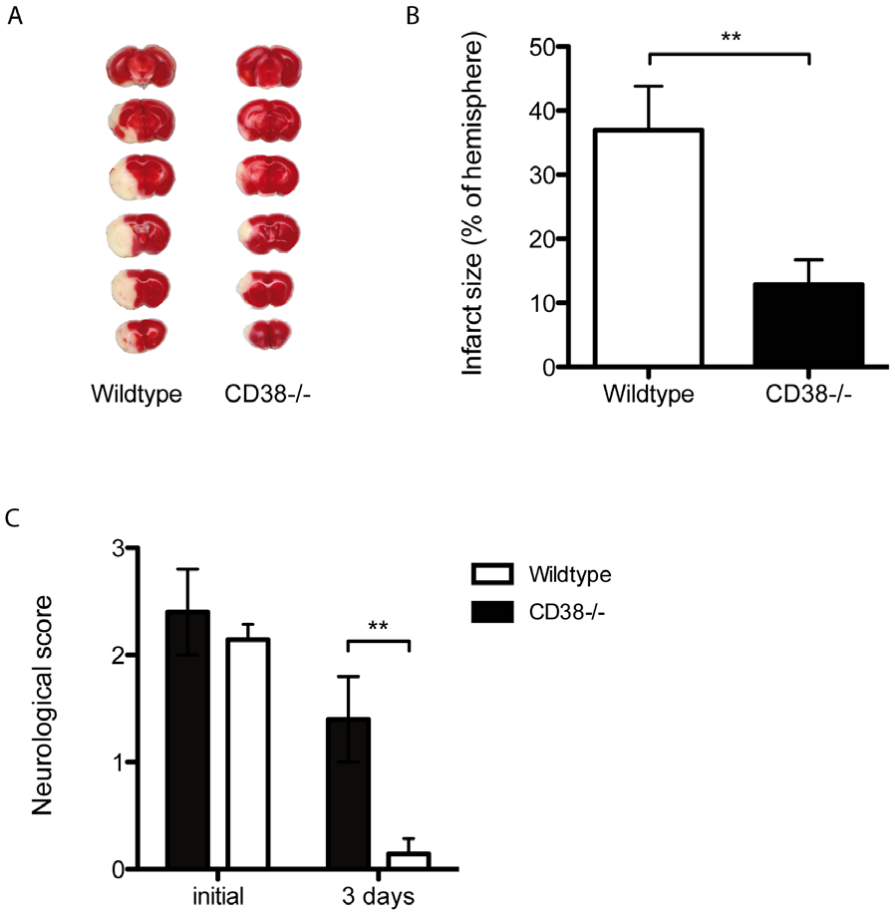
CD38-deficiency protects from ischemic stroke and ameliorates clinical impairment after tMCAO. Coronal sections of representative mouse brains were prepared 3 days after tMCAO from wildtype and CD38^−/−^ mice. Vital tissue stains red after treatment with TTC. Infarct size of CD38^−/−^ mice was significantly decreased compared to wildtype mice (A). Corresponding to the cerebral infarct size, neurological scores in CD38^−/−^ mice were significantly lower three days after tMCAO compared to wildtype indicating an attenuated clinical impairment (B) (n = 5–7; *p<0.05 versus).

Finally, focal ischemic infarct size was analyzed after tMCAO for one hour with the intraluminal filament technique in wildtype and CD38^−/−^ animals. Coronal sections were collected after a 3-day reperfusion period and stained with 2,3,5-triphenyltetrazolium chloride. The ischemic area in CD38^−/−^ mice was significantly decreased to 12.8±4% of hemisphere as compared to 37.0±7% of hemispheric volume in control mice (figure 4A). One hour after MCAO, neurological scores were comparable (control 2.4±0.4, n = 5; CD38^−/−^ 2.1±0.1, n = 7, figure 4B). After three days CD38^−/−^ mice showed only minimal neurological deficit, while neurological impairment was significantly increased in control mice (control 1.4±0.4, n = 5; CD38−/− 0.1±0.1, n = 7, figure 4C).

## Discussion

Increasing evidence indicates that the postischemic inflammatory response might play a detrimental role in the secondary progression of stroke injury [2]. Immune-deficient animal models revealed an amelioration of cerebral ischemic-reperfusion injury [12], [18], [19]. The inflammatory infiltrates and its mainly cytotoxic processes after cerebral ischemia seem to be local and contained to the penumbral region [14]. Therefore, the recruitment and accumulation of leukocytes play pivotal roles in initiating and mediating the cerebral inflammation. Our current work suggests that CD38 influences immune cell migration as well as activation, which are both required for the mainly detrimental postischemic inflammatory response contributing to secondary brain damage.

In CD38-deficient mice, the accumulation of T-cells and especially macrophages was decreased whereas the amount of microglia did not differ significantly (figure 2). Two mechanisms could be accounted for this - i.e. impaired chemokinesis and/or decreased chemoattraction. Especially, the chemotaxis of myeloid immune cells, like myeloid dendritic cells and neutrophils, highly depends on CD38 and its products ADPR and cADPR [9], [10]. Therefore, it is not surprising that CD38^−/−^ mice show a twenty fold lower number of infiltrating macrophages than wildtype mice, but only a three fold lower numbers of infiltrating T-cells (figure 2A and B). Experiments with GFP^+^ bone marrow chimera confirm previous observations, macrophages and myeloid dendritic cells found in the ischemic hemisphere, originate in the bone marrow, while microglia are resident and evolve independent of bone marrow stem cells (figure S2). Therefore, CD38 differentially influences the migration of hematopoetic myeloid immune cells.

In addition to an impaired migratory potential, a weaker induction of local MCP-1 production was observed in CD38^−/−^ than in wildtype mice after stroke, despite a similar initial ischemic brain damage indicated by comparable neurological impairment and similar elevations of MCP-1 levels in peripheral blood 6 h after tMCAO illustrating adequate systemic immune responses. Furthermore, unaltered levels of TNF-α and INF-γ in the ischemic hemisphere demonstrate a distinct attenuation of MCP-1 expression after stroke (figure 1). Among all pro-inflammatory chemokines, MCP-1 dependent migration is known to strongly rely on CD38 as well as its products ADPR and cADPR [10]. Importantly, the interaction of MCP-1 with its receptor CCR2 has been attributed a central role in experimental cardiac, renal and cerebral ischemia-reperfusion models [20]. After focal cerebral ischemia an early and local production of MCP-1 was described in rat, mouse and human patients [21], [22], [23], [24]. Previous studies have shown that genetic ablation of MCP-1 or its receptor CCR2 resulted in reduced cerebral injury closely related with an attenuated accumulation of monocytes and macrophages after stroke [3], [4]. In contrast, focal MCP-1 overexpression in brain exacerbated the cerebral infarct volume and was associated with increased local transmigration and perivascular accumulation of macrophages after ischemic stroke [25]. Nonetheless, decreased MCP-1 production will attenuate attraction and recruitment of monocytes and myeloid dendritic cells and subsequently ameliorate the post-ischemic inflammatory response. Possibly, macrophages are necessary to sustain this mainly detrimental immune reaction, because macrophages themselves represent a significant source for pro-inflammatory cytokines and chemokines, like MCP-1 [26], [27], [28]. Furthermore, the accumulation of macrophages can be observed as early as six hours after tMCAO and corresponds well with an up-regulation of CD38 and therefore immune cell activation [14]. In contrast, no significant alteration of CD38 expression was observed in microglia, CD4^+^ cells and myeloid dendritic cells (figure 3). In line with previously published work, migration, attraction and activation of macrophages are critical steps for the initiation and preservation of pro-inflammatory immune processes after focal cerebral ischemia. Our data show that these processes highly depend on CD38, because macrophages strongly up-regulate CD38 upon stroke and highly rely on CD38 for effective migration into the ischemic brain. Therefore macrophages could be the key player to orchestrate the CD38-dependent effects, because they represent one of the earliest cell populations to infiltrate the ischemic brain, up-regulate CD38 after stroke and are known to be a major source of MCP-1 production. Nevertheless decreased macrophage infiltration could merely be a consequence of reduced infarct volume. But in this case, the amount of microglia would also be reduced, because the inflammation-independent ischemic brain damage would alter the amount of all immune cell infiltrates proportionally.

In summary, our data suggest that attenuation of postischemic cerebral inflammatory processes decrease secondary neurotoxic cell death, decrease infarct size and ameliorate clinical neurological impairment. We have shown that CD38 orchestrates the recruitment and activation of immune cell subpopulations, the production of pro-inflammatory cytokines and cytotoxic autoimmune response after stroke. Therefore, CD38 might prove to be a therapeutic target to modulate the inflammatory mechanisms after cerebral ischemia.

## Supporting Information

Figure S1 **Exemplary gating strategy for infiltrating immune cells**. Gating strategy for a cell subset derived from 3 days postreperfusion ipsilesional stroked hemispheres (A). Populations were gated back onto initial CD45/SSCplot (B). Yellow microglia, green neutrophils, red and orange DCs, and macrophages, black and purple lymphocytes, and NK cells.
(TIFF)

Figure S2 **Macrophage infiltration is attenuated in CD38−/− compared with wildtype mice.** Staining for GFAP to demarcate the infarct zone reveals reduced infarct sizes in CD38^−/−^ compared with wildtype 3 days after MCAO for one hour (A, F). Furthermore, the reduced amount of CD11b^+^ monocytes (B, C for wildtype, G, H for CD38^−/−^) could be attributed to macrophages, which were distinguished by isolectin^+^ staining and morphology (see arrow for microglia morphology; B, D for wildtype, G, I for CD38^−/−^). The overall amount of cells was similar as visualized by DAPI staining (E, J).
(TIF)

Figure S3 **Macrophages and myeloid dendritic cells infiltrate the ischemic brain, whereas microglia expand locally.** Irradiated wildtype mice reconstituted with GFP positive bone marrow cells were subjected to 1h tMCAO. After three days of reperfusion immune cells were isolated and GFP expression of different subtypes was discriminated by FACS-analysis Macrophages (dark grey) and myeloid dendritic cells (light grey) highly expressed GFP and therefore originated from the reconstituted bone marrow. In contrast, microglia (black) were resident (n = 3 with four animals for each experiment).
(TIF)
