# Correction: CD38 Exacerbates Focal Cytokine Production, Postischemic Inflammation and Brain Injury after Focal Cerebral Ischemia

**DOI:** 10.1371/annotation/295c388d-013d-4bb9-b4e4-da8e88317594

**Published:** 2011-07-28

**Authors:** Chi-un Choe, Kerstin Lardong, Mathias Gelderblom, Peter Ludewig, Frank Leypoldt, Friedrich Koch-Nolte, Christian Gerloff, Tim Magnus

The black and white fill in Figure 4c is incorrect. Please view the correct Figure 4 here: ttp://www.plosone.org/corrections/pone.0019046.g004.cn.tif

