# Supplementary figures and images for: CD38 Exacerbates Focal Cytokine Production, Postischemic Inflammation and Brain Injury after Focal Cerebral Ischemia
Source: PLoS One. 2011 May 13;6(5):e19046. doi: 10.1371/journal.pone.0019046 (PMC3097994; doi:10.1371/journal.pone.0019046)

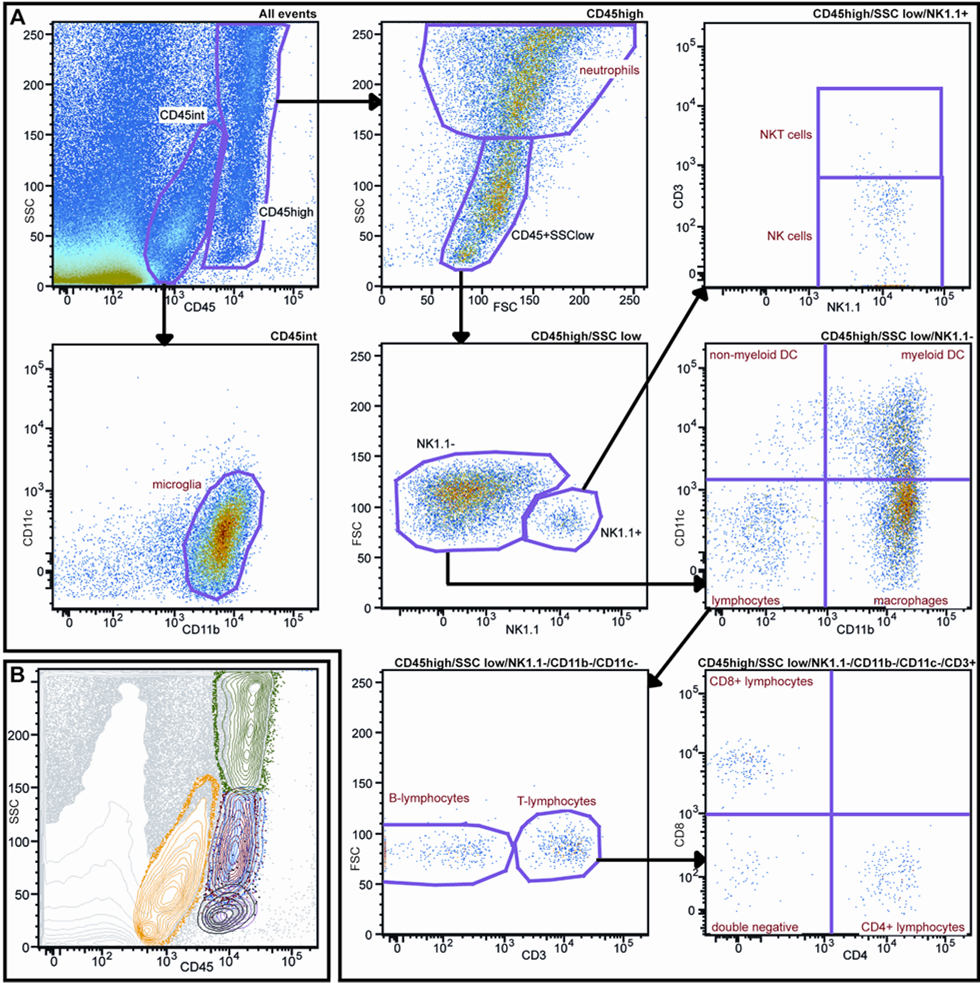

Supplement: Figure S1 — Exemplary gating strategy for infiltrating immune cells. Gating strategy for a cell subset derived from 3 days postreperfusion ipsilesional stroked hemispheres (A). Populations were gated back onto initial CD45/SSCplot (B). Yellow microglia, green neutrophils, red and orange DCs, and macrophages, black and purple lymphocytes, and NK cells. (TIFF) [file pone.0019046.s001.tiff]

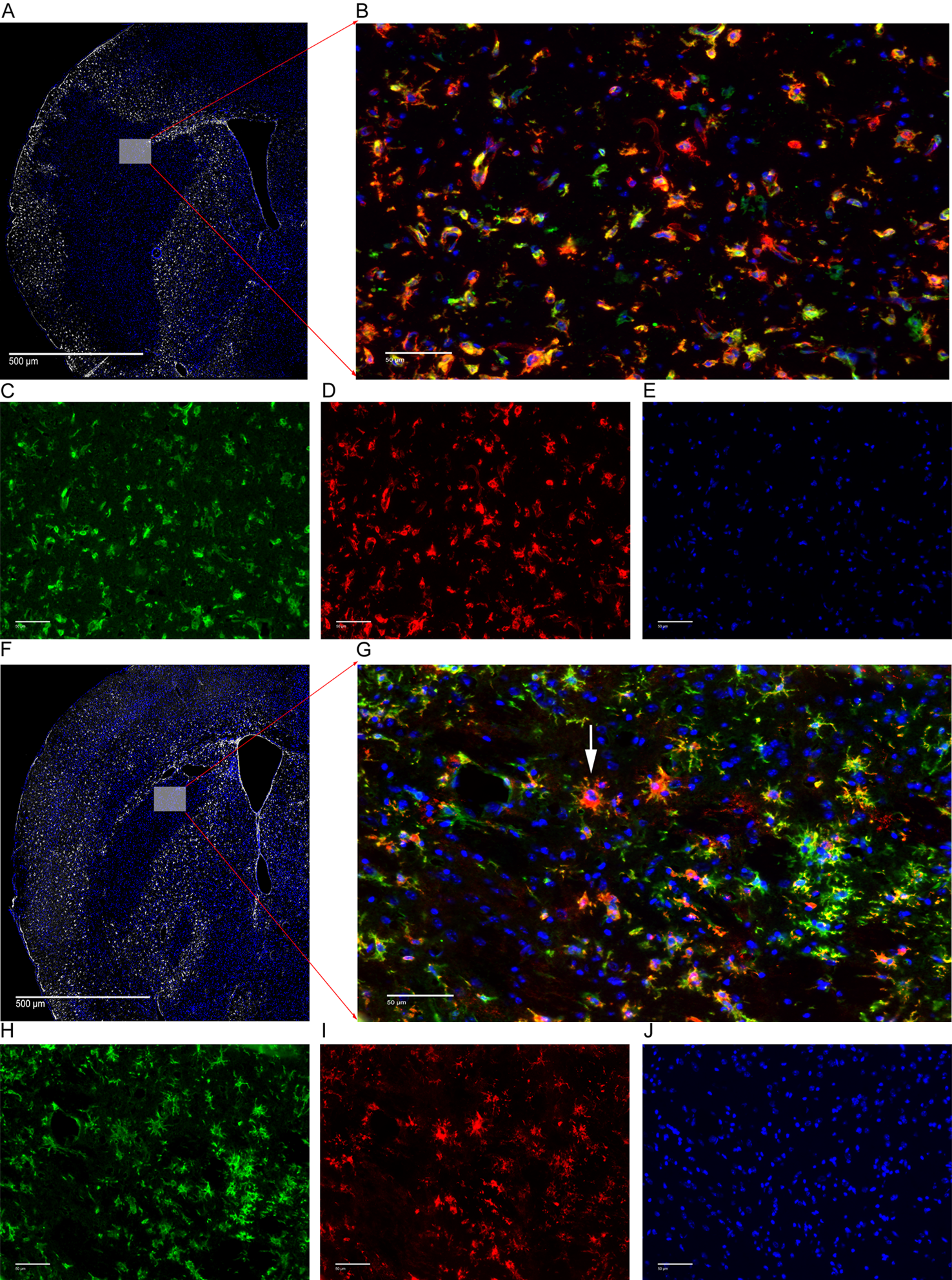

Supplement: Figure S2 — Macrophage infiltration is attenuated in CD38−/− compared with wildtype mice. Staining for GFAP to demarcate the infarct zone reveals reduced infarct sizes in CD38−/− compared with wildtype 3 days after MCAO for one hour (A, F). Furthermore, the reduced amount of CD11b+ monocytes (B, C for wildtype, G, H for CD38−/−) could be attributed to macrophages, which were distinguished by isolectin+ staining and morphology (see arrow for microglia morphology; B, D for wildtype, G, I for CD38−/−). The overall amount of cells was similar as visualized by DAPI staining (E, J). (TIF) [file pone.0019046.s002.tif]

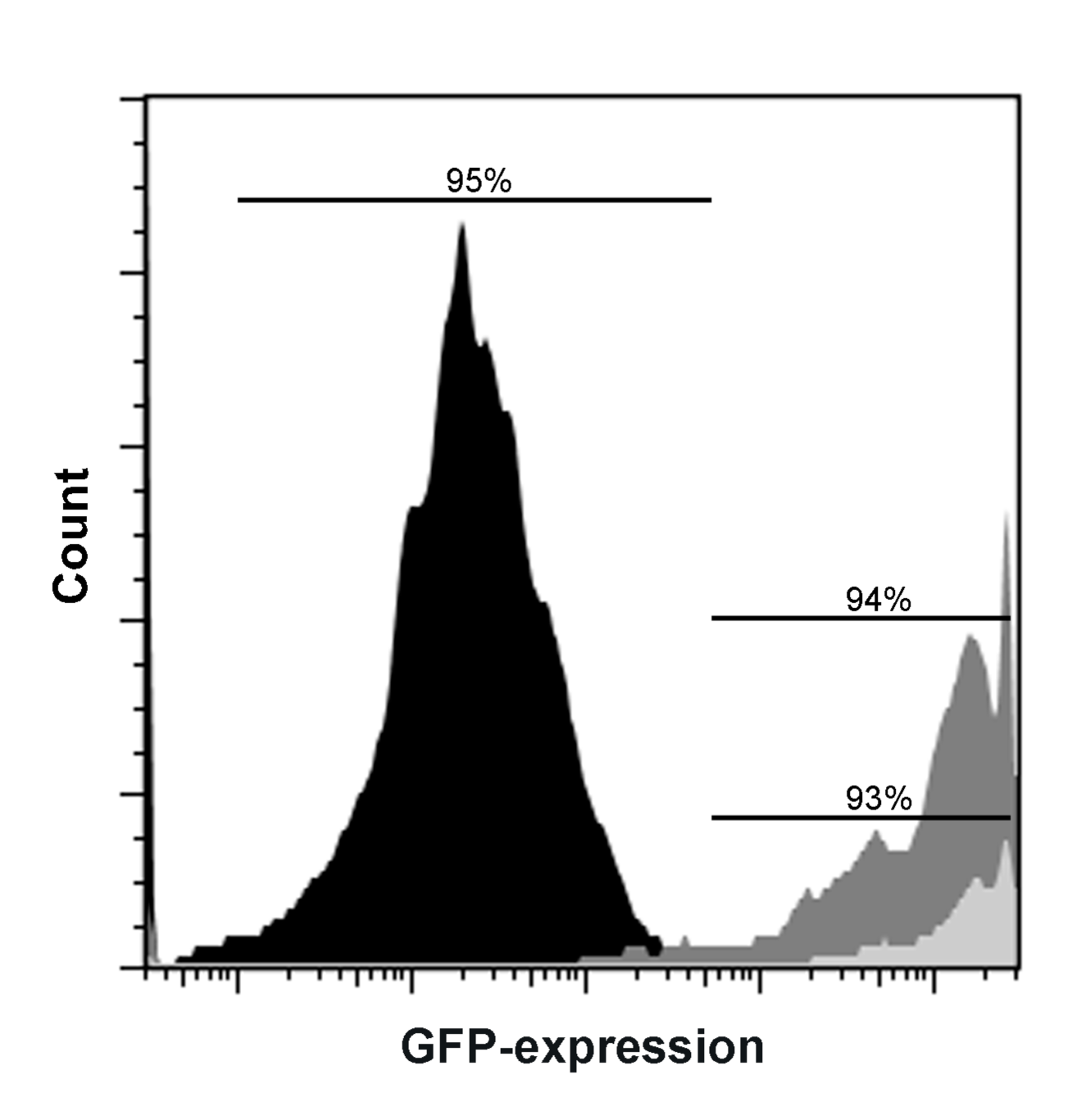

Supplement: Figure S3 — Macrophages and myeloid dendritic cells infiltrate the ischemic brain, whereas microglia expand locally. Irradiated wildtype mice reconstituted with GFP positive bone marrow cells were subjected to 1h tMCAO. After three days of reperfusion immune cells were isolated and GFP expression of different subtypes was discriminated by FACS-analysis Macrophages (dark grey) and myeloid dendritic cells (light grey) highly expressed GFP and therefore originated from the reconstituted bone marrow. In contrast, microglia (black) were resident (n = 3 with four animals for each experiment). (TIF) [file pone.0019046.s003.tif]
